# The Movement Ecology of the Straw-Colored Fruit Bat, *Eidolon helvum*, in Sub-Saharan Africa Assessed by Stable Isotope Ratios

**DOI:** 10.1371/journal.pone.0045729

**Published:** 2012-09-21

**Authors:** Gonzalo Ossa, Stephanie Kramer-Schadt, Alison J. Peel, Anne K. Scharf, Christian C. Voigt

**Affiliations:** 1 Leibniz Institute for Zoo and Wildlife Research, Berlin, Germany; 2 Department of Veterinary Medicine, University of Cambridge, Cambridge, United Kingdom; 3 Institute of Zoology, Zoological Society of London, Regent’s Park, London, United Kingdom; 4 Behavioral Biology, Freie Universität Berlin, Germany; Max Planck Institute for Evolutionary Anthropology, Germany

## Abstract

Flying foxes (Pteropodidae) are key seed dispersers on the African continent, yet their migratory behavior is largely unknown. Here, we studied the movement ecology of the straw-colored fruit bat, *Eidolon helvum*, and other fruit bats by analyzing stable isotope ratios in fur collected from museum specimens. In a triple-isotope approach based on samples of two ecologically similar non-migratory pteropodids, we first confirmed that a stable isotope approach is capable of delineating between geographically distinct locations in Sub-Saharan Africa. A discriminant function analysis assigned 84% of individuals correctly to their capture site. Further, we assessed how well hydrogen stable isotope ratios (δ^2^H) of fur keratin collected from non-migratory species (n = 191 individuals) records variation in δ^2^H of precipitation water in sub-Saharan Africa. Overall, we found positive, negative and no correlations within the six studied species. We then developed a reduced major axis regression equation based on individual data of non-migratory species to predict where potentially migratory *E. helvum* (n = 88) would come from based on their keratin δ^2^H. Across non-migratory species, δ^2^H of keratin and local water correlated positively. Based on the isoscape origin model, 22% of *E. helvum* were migratory, i.e. individuals had migrated over at least 250 km prior to their capture. Migratory individuals came from locations at a median distance of about 860 km from the collection site, four even from distances of at least 2,000 km. Ground-truthing of our isoscape origin model based on keratin δ^2^H of extant *E. helvum* (n = 76) supported a high predictive power of assigning the provenance of African flying foxes. Our study highlights that stable isotope ratios can be used to explain the migratory behavior of flying foxes, even on the isotopically relatively homogenous African continent, and with material collected by museums many decades or more than a century ago.

## Introduction

Flying foxes (family Pteropodidae) are central ecosystem service providers in the Old World [1], [2]. More than 280 plant species benefit from flying foxes either by pollination and/or seed dispersal. Ecosystem services offered by pteropodids result in about 450 economically valuable plant products like fruits or timber [1], [3], [4]. Flying foxes are essential for maintaining the genetic connectivity of plants among fragmented patches of rainforests and distant habitats due to their capability to move over long distances [5]–[7]. Pteropodids play also a prominent role as a reservoir for zoonotic diseases [8]–[11]. Thus, understanding the movement ecology of pteropodids is important, not only from a conservation point of view but also from an epidemiological and human health standpoint. In this study, we looked at the movement ecology of *Eidolon helvum* to better understand their potential role in the large-scale distribution of seeds across the African continent and to shed light on the spatial use of flying foxes in context to zoonotic diseases. Secondly, we tested for the first time whether variation in hydrogen stable isotope ratios of fur keratin can be used to assess the origin of flying foxes in Africa.

The primary habitat of *E. helvum* encompasses the tropical forests and savannahs in central Africa, from where it migrates following seasonal fluctuations in precipitation and associated changes in food abundance [12]. This species provides important services of pollination and promotes out-crossing in flowering plants [13]. For example, Daïnou and colleagues [14] suggested that migratory movements of *E. helvum* largely affect the geographic distribution of *Milicia excelsa* (Moraceae), one of the most important sources for timber in Africa [15]. Baker highlighted the ecological importance of *E. helvum* for West African ecosystems by cross-pollinating *Ceiba pentandra* (Malvaceae) [16]. Due to the large population sizes, *E. helvum* is potentially a key species for regenerating African forests [17]. Populations of *E. helvum* seem to follow the seasonal variations in fruit abundance [18], [19], i.e. migrating along a south – north axis in West Africa, from Ivory Coast to Niger. Other studies describe specific sedentary periods during a year when individuals aggregate in large colonies [20]–[22]. Most recent studies using satellite telemetry in three individuals at the Kasanka National Park in Zambia showed that *E. helvum* is capable of migrating several thousands of kilometers in a period of five months [5]. Here, we used a novel approach to the study of migration in *E. helvum* of sub-Saharan Africa by analyzing stable isotope ratios in fur collected from museum specimens and from live animals in their natural habitat.

Hydrogen stable isotope ratios have been increasingly used as endogenous markers to study the movement ecology of birds [23], [24] and bats [25]–[27]. The major advantages of this approach are that the movement ecology of animals can be assessed from a single capture event [28], [29], and that even historical specimens from museum collections can be studied. The stable isotope approach in the study of animal migration is based on the following two premises: (1) Stable hydrogen isotope ratios of rainfall vary geographically according to temperature [30]–[32]. This is because when gaseous water travels in the atmosphere along temperature gradients, water molecules labeled with heavy isotopes condensate faster than those labeled with light isotopes [33]. (2) Biologically inert body products, such as feather and fur, retain the stable isotope signature of the location where they grew [25], [26], [28], [29]. Recently, other elements such as carbon and nitrogen have been included in studies to improve the geographical resolution for predicting the provenance of animals [26], [34]. Environmental variation in carbon stable isotopes is usually related to geographic variation in plant species compositions and their specific photosynthetic metabolism of CO_2_ fixation (C3, C4 and CAM). Nitrogen isotope ratios of ecosystems are known to vary with water availability [35], and, therefore, recent studies included nitrogen as well in triple-isotope approaches for geolocating animals, e.g. European bats [26] or Afrotropical birds [34].

In this study, we wanted to elucidate the migratory behavior of the straw-colored fruit bat, *E. helvum*, in sub-Saharan Africa based on fur material from museum specimens collected over the course of the last century. We organized our study in three parts. Firstly, we used a triple-isotope approach (carbon, nitrogen and hydrogen) to assess whether fur keratin varies geographically in non-migratory flying foxes in general, and whether geographically distinct populations can be separated based on stable isotope of these three elements. We then focused on hydrogen stable isotope ratios, because currently we lack a geographically complete isoscape for nitrogen and carbon on the African continent; a prerequisite for following a triple-isotope approach. Secondly, we tested within each and across non-migratory species whether hydrogen stable isotope ratios of fur keratin records variation of hydrogen stable isotope ratios extrapolated for precipitation water of the sites where individuals were collected for museums. Based on data of these non-migratory species, we developed a reduced major axis (RMA) regression model. Thirdly, using the RMA regression model, we assessed the likely migratory movements of *E. helvum* specimens in the period prior to their collection. This assessment was based on information of a) site of capture, b) hydrogen stable isotope ratios of fur keratin in museum specimens, and c) the extrapolated hydrogen isoscape of the African continent [31]. Lastly, we tested the quality of our isoscape origin model by applying it to data obtained from contemporary *E. helvum* individuals captured in two Tanzanian cities. We preferred to validate our approach with live animals over museum specimens, because we obtained better information about the time and exact location of capture for contemporary bats than for specimens of museum collections. Here, we show that stable isotope ratios can be used in the study of migratory vertebrates even in the relatively homogenous isoscape of the African continent. Further, we show that large portions of *E. helvum* populations are non-migratory in Sub-Saharan Africa, but that some individuals can travel as far as several thousand kilometers. This supports the potential of *E. helvum* for providing long-distance seed dispersal across the African continent.

## Materials and Methods

### Selection of Species and Collection of Fur Material from Museums

In our study, we focused on *E. helvum* (n = 88 individuals) which is assumed to migrate over large parts of the sub-Saharan African continent, and as a reference we selected six likely non-migratory pteropodid species: *Rousettus aegyptiacus* (n = 34 individuals), *Lissonycteris angolensis* (n = 17), *Epomophorus wahlbergi* (n = 23), *Hypsignathus monstrosus* (n = 30), *Epomops franqueti* (n = 30), and *Epomophorus crypturus* (n = 57). Non-migratory species were selected so to maximize sample size for each species, based on those available in the collections of the Natural History Museum in Paris (MNHN Paris), France, and of Berlin (MfN Berlin), Germany. Since past studies on pteropodids provided only very limited insights into the movement ecology of these species [36], we were not able to choose a priori bat species with documented non-migratory habits, except *R. aegyptiacus* and *L. angolensis*. These two species are known to roost in cave shelters during daytime and are considered to be strictly sedentary [37]. Daily foraging movements of *R. aegyptiacus* are usually limited to less than 50 km [7], yet one study suggests long-distance migration in a single individual of *R. aegyptiacus* [38]. Both species have a broad but patchy distribution in sub-Saharan Africa, probably related to the patchy distribution of cave roosts across the African continent [36]. *E. wahlbergi* and *H. monstrosus* perform only short distance movements of a few kilometers [39], [40]. We lack any information about the movement ecology of *E. franqueti*, a pteropodid with a wide distributional range from Senegal to Ethiopia [41]. *E. crypturu*s is considered to travel “considerable distances” in search for food, yet more accurate descriptions of the exact distances and whether movements are performed seasonally are lacking [41]. The target species of this study, *E. helvum*, is classified as Near Threatened by the International Union for Conservation of Nature (IUCN) in response to the considerable decline of local populations due to hunting for food, use in traditional medicine and owing to the large-scale deforestation in many areas of its distributional range [42]. African populations of *E. helvum* are classified in the appendix II of the Convention on Migratory Species [43], according to their broad distribution in sub-Saharan Africa, and their ecological service function by pollination and seed dispersal [43]. Since there is currently no indication for a gender-specific migratory behavior in *E. helvum*, we did not differentiate between males and females in our analysis.

We obtained fur samples from alcohol preserved specimens from the two museums (Table 1) that came from collections conducted over a period of more than 100 years (1881–2011; Table S1). Allowance to collect fur samples from museum specimens was granted by the responsible mammal curator; Specimens remained in the corresponding collection during and after sample collection. We chose specimens for which information about the location and preferentially the year of collection was available. The locations of bats included in this study are given in the electronic supplementary material (Table S1, Figure S1). From each of the selected specimens we collected a small fur samples (about 2 mg) from the inter-scapular region of the bats’ back. Samples were placed in plastic tubes (0.5 ml), marked with the serial number of the respective museum, stored at ambient temperature and shipped to the stable isotope laboratory of the Leibniz Institute for Zoo and Wildlife Research (IZW). We determined the geographic coordinates and altitude for the collection localities using Google Earth [44].

**Table 1:** Number of fur samples collected from museum specimens of seven species of Pteropodidae according to museum origin.

Species	MNHNParis	MFNBerlin	Totaln	δ^2^H_K_(‰)	δ^ 13^C(‰)	δ^15^N(‰)
*Eidolon helvum*	55	33	88	−89.1±14.0	−21.8±1.0	8.0±1.2
				(−160.0; −61.6)	(−24.5; −19.5)	(4.9; 11.0)
*Epomophorus crypturus*	57	–	57	−72.4±8.2	−20.5±0.9	8.4±2.5
				(−107.4; −65.6)	(−23.5; −21.0)	(3.9; 13.6)
*Epomophorus wahlbergi*	4	19	23	−83.2±8.6	−21.9±0.4	7.1±1.5
				(−97.6; −62.4)	(−22.8; −18.3)	(6.1; 18.3)
*Epomops franqueti*	–	30	30	−89.5±9.7	−22.2±0.6	6.8±2.5
				(−105.9; −62.6)	(−27.4; −19.4)	(4.0; 12.5)
*Hypsignathus monstrosus*	–	30	30	−88.5±6.0	−22.8±1.1	6.5±0.8
				(−101.4; −65.1)	(−22.6; −20.9)	(4.9; 10.4)
*Lissonycteris angolensis*	2	15	17	−75.9±14.0	−21.7±1.0	8.7±2.5
				(−100.8; −75.1)	(−25.6; −20.3)	(4.7; 7.8)
*Rousettus aegyptiacus*	13	21	34	−78.0±7.2	−20.7±0.7	8.2±1.5
				(−96.6; −63.7)	(−22.4; −19.1)	(6.5; 13.0)
Total samples	131	148	279			

Mean values (±one SD) and the range (minimum and maximum values) of hydrogen (δ^2^H), carbon (δ^13^C) and nitrogen (δ^15^N) stable isotope ratios in fur samples of the selected species.

### Collection of Fur Material from Live Specimens for Model Validation

In addition to the samples from museum specimens, we also collected 76 fur samples of extant *E. helvum* from two colonies in Tanzania: 44 samples from a colony in Dar es Salaam (S 06.80134, E 039.28249) and 32 from Morogoro (S 06.80134, E 039.28249). Bats were captured using mistnets between 28^th^ of August and 29^th^ of September 2009 and fur samples were collected from the interscapular region of the dorsal fur, transferred to plastic vials and shipped to the IZW. Local residents reported that the *E. helvum* colonies were present year-round; yet it is unknown whether migratory individuals may join the non-migratory population at certain times. Migration has been reported to occur between October and December in East Africa [18]. Since we collected samples in August and September, we categorized the captured animals as non-migratory. Collection of samples from extant *E. helvum* was granted by the Tanzanian authorities (COSTECH permit: 2009-225-NA-2009-160). Prior to this, sample collection was approved by the Animal and Care and Ethics Committee of the IZW. All bats were released at the site of capture after fur collection.

### Hydrogen Stable Isotopic Analysis

All analyses of hydrogen, carbon and nitrogen stable isotope ratios were carried out at the Stable Isotope Laboratory of the IZW (Berlin, Germany). Fur samples were cleaned with 2∶1 chloroform methanol solution for 24 hours to remove dirt and surface oils. The solvent was discarded, and the tubes with fur samples deposited in a drying oven (Heraeus T6, Thermo Electronic Corporation, Bremen, Germany) at 50°C for at least one day to eliminate the remainder of the solvent. We analyzed the hydrogen stable isotope ratio of samples in sequence with keratin reference material of known isotope ratio as described by [45]. This method controls for the exchange of hydrogen in keratin with ambient air. Thus, we refer to the non-exchangeable portion of hydrogen in fur keratin in the remainder of the text. The references used were powdered sheep fur from Sweden (SWE-SHE; 0.250±0.01 mg; mean ± one standard deviation) and from Spain (ESP-SHE; 0.249±0.01 mg) and goat fur from Tanzania (AFR-GOA; 0.255±0.01 mg). Each of these references was obtained from a single domestic animal (sheep or goat). The stable isotope ratios of the non–exchangeable hydrogen of the reference samples were −167.9±1‰ (SWE-SHE; (mean ± one standard deviation), −108.3±1‰ (ESP-SHE) and −66.3±0.9‰ (AFR-GOA) [26], [46].

From each of the fur and reference samples, we transferred a subsample of on average 0.274±0.01 mg into 3.5×5.0 mm silver foil capsules (OEA Laboratories, Callington, Cornwell, UK). Loaded capsules were formed as small cubes, transferred into a 96 port microtiter tray (69 bat fur subsamples +27 references of keratin hydrogen isotope) and stored in a drying oven at 50°C for at least 24 hours to allow subsamples and references to equilibrate with ambient air. Loaded capsules were then transferred to an autosampler carousel (Zero Blank autosampler – Costech Analytical Technologies Inc. Italy; [26], [47]).

Samples were pyrolized in a high-temperature elemental analyzer at 1350°C (HTO Elementaranalysator, HEKAtech GmbH, Wegberg, Germany). The resulting gases were carried by chemically pure helium (Linde, Luna, Germany) of H_2_, N_2_ and CO (N_2_ and CO are sub products) were separated in a chromatographic column at 80°C. Hydrogen was carried to an isotope ratio mass spectrometer (Delta V Advantage IRMS, ThermoFisher Scientific, Bremen, Germany). All hydrogen stable isotopic ratios were expressed using the δ notation as units per mill (‰) and normalized on the Vienna Standard Mean Ocean Water (V-SMOW) standard scale. The precision of repeated measurements of laboratory standards was better than 1.2 ‰ (one Standard deviation) for hydrogen. In the remainder of the text, we will refer to the hydrogen stable isotope ratio of the non-exchangeable portion as δ^2^H_K_ and to the hydrogen stable isotope ratio of extrapolated precipitation water as δ^2^H_P_. To obtain the mean annual δ^2^H_P_ from precipitation for each locality [31], we used the raster dataset in ArcGrid format provided by Bowen [46].

### Carbon and Nitrogen Stable Isotope Analysis

For carbon and nitrogen stable isotope ratios, we used protein laboratory standards as references (Tyrosin: Tyr-b1; 0.35–0.45 mg; Leucine: Leu-b1; 0.25–0.30 mg) to monitor possible instrumental drift. From each laboratory standard and fur sample, we placed subsamples of 0.40±0.05 mg into 3.5×5.0 mm in tin foil capsules (OEA Laboratories, Callington, Cornwall, UK), and then transferred these packages to the autosampler carousal (MAS 200 autosampler, Thermo Electron Corporation, Bremen, Germany). Samples were oxidized in the reactor of the elemental analyzer (Flash EA 1112, Thermo Electron Corporation, Bremen, Germany). The resulting gaseous products were separated into CO_2_ and N_2_. The ratios of the stable isotopes ^13^C/^12^C and^ 15^N/^14^N of the resulting organic combustion were measured using a Delta V Advantage isotope ratio mass spectrometer (Thermo Finnigan, Bremen, Germany) and were reported in δ notation in units per mill (‰) relative to the international Vienna-PeeDeeBelemnite (V-PDB) or ambient nitrogen (AIR-N_2_) standard respectively. Laboratory standards were previously calibrated with standards (NBS 19, NBS 22, USGS 24 and L-SVEC for carbon; IAEA-N-1, IAEA-N-2 and IAEA-NO-3 for nitrogen). The δ^13^C and δ^15^N values of the laboratory standards were −24.0‰ and 4.4‰ respectively for Tyr-b1 and −30.3‰ and 11.0‰ respectively for Leu-b1. The precision of repeated measurements of laboratory standards was better than 0.18 ‰ (one standard deviation) for nitrogen and 0.14 ‰ (one SD) for carbon.

### Statistical Analyses

All statistical analyses were performed with R 2.14.2 [48], and the significance values of all tests were set at 0.05. Values are presented as means ± one standard deviation unless stated otherwise. We tested for a normal distribution of data using the Shapiro-Wilk normality test and checked for heterogeneity of variances by plotting the data. In order to meet the assumptions of linear models, we excluded outliers with extreme values.

In our triple-isotope approach, we studied the explanatory power of δ^2^H, δ^13^C and δ^15^N to delineate between populations of non-migratory species from various geographical locations [26]. We focused on two species, *E. crypturus* and *E. wahlbergi*, that were similar in their ecology and were most likely to be non-migratory [49]. We only included data in our analysis that originated from locations where at least three individuals had been collected. To test for differences in δ^2^H_K,_ δ^13^C_,_ and δ^15^N among localities, we used a multivariate analysis of variance (MANOVA). Then, we used a discriminant function analysis (package *MASS*) to determine if site assignment was improved using the triple-isotope approach and by determining the proportion of correct assignments.

To assess the predictive power of hydrogen stable isotope ratios in fur keratin of non-migratory species for localizing species’ origins, we compared δ^2^H_K_ with predicted δ^2^H_P_ (obtained from http://www.waterisotopes.org). Since we usually lacked information about the Julian date of specimen collection, we used the annual average of δ^2^H_P_ for the collecting sites. We calculated Pearson’s product moment correlation (*r*) for the relationship between δ^2^H_K_ of non-migratory species and δ^2^H_P._

We calculated a reduced major axis (RMA) regression model based on the data of the non-migratory species to assess the movement ecology of *E. helvum.* For this, we linked δ^2^H_K_ of non-migratory specimens (n = 140) and annual mean δ^2^H_P_ values in Africa [45] (package *lmodel2*, [50]) to account for errors in both measures. Once the δ^2^H_k_ values were translated into δ^2^H_P_, we modeled the uncertainty related to the analytical error using the δ^2^H_K_ standard deviations (σ_location_) of the bat populations of known origin (non-migratory species) included in the regression model as error estimate and fitted a gamma distribution Γ to σ_location_ using maximum likelihood. We fitted Γ to 20 values of σ_location_, having a minimum of 3 samples for each location. The shape of this function was k = 4.13 and the scale (θ) was 1.64.

To assess the migratory behavior of flying foxes based on stable isotope ratios measured in museum specimens, we modeled the origin of *E. helvum* using their individual δ^2^H_K_ values, as outlined in [27] and modified from [29] where each value of δ^2^H_K_ was associated to a location according to the African isoscape of hydrogen [45]. We reproduced the error of the distribution by a simulation of 5,000 possible values of δ^2^H_P_ for each individual’s δ^2^H_K_ drawing randomly from a normal distribution N(μ, σ), where μ corresponds to the exact value of δ^2^H_P_ obtained from the RMA regression, and σ was randomly generated from the gamma distribution Γ. From this, we created a probability density map using a probability density function (PDFs, package *MASS*) for each migratory individual (i.e. 88 maps for *E. helvum*). PDFs for each individual were standardized to range between 0 and 1. Using an ASCII remap file for each individual, we created the final map of origin for each individual. The reclassified raster surface was mapped using ArcGIS v. 9.3.1 with a 10′×10′ (arc minutes) resolution; distribution range of *E. helvum* was downloaded from the IUCN Red List of Threatened Species (www.iucnredlist.org). For each individual, we visually selected the maps showing the same origin probability (i.e. maps corresponding to individuals with similar δ^2^H_K_ from fur samples) and then calculated a mean probability map using the raster calculator from ArcGIS v. 9.3.1. For each mean probability map, we measured the distance of collection site of individuals to the nearest pixel with a minimum probability of origin of 0.7 inside the IUCN distribution range of the species, i.e. we considered probability values higher than 70% to be of sufficient discriminatory power.

We validated the isoscape origin model using stable hydrogen isotope ratios measured in fur keratin of contemporary *E. helvum*. The data of these live non-migratory individuals were not used in the RMA regression. We performed the same spatial analysis as described above based on annual δ^2^H_P_ values. We preferred the data set of annual δ^2^H_P_ over monthly δ^2^H_P_, since we planned to validate the isoscape origin model that was based on annual δ^2^H_P_. Lastly, we calculated the probability that colony members originated from the two collection sites based on the isoscape origin model.

## Results

### A Triple-isotope Approach to Geolocate Flying Foxes in Africa

**Figure 1:**
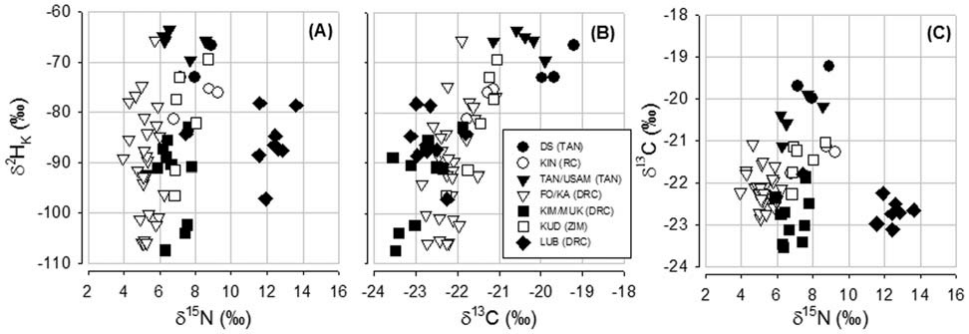
Relationships between stable isotope ratios of carbon, nitrogen and hydrogen measured in fur keratin of *Epomophorus wahlbergi* and *E. crypturus*. (**A**) Relationship between hydrogen and nitrogen stable isotope ratios, (**B**) relationship between hydrogen and carbon stable isotope ratios, (**C**) relationship between carbon and nitrogen stable isotope ratios. Each symbol represents one individual. Localities are indicated according to the legend. Abbreviations: DS = Dar es Salaam (Tanzania), KIN (RC) = Kinshasa (Republic of Congo), TAN/USAM (TAN) = Tanga, Usambara (Tanzania), FO/KA = Forzt/Kaloba (Democratic Republic of Congo), KIM/MUK (DRC) = Kimbongo/Mukulakula (Democratic Republic of Congo), KUD (ZIM) = Kudzwe (Zimbabwe), LUB = Lubumbashi (Democratic Republic of Congo).

The sampling localities of the 279 fur samples analyzed from African pteropodid bats ranged between 18.08° and −34.36° in latitude, between −40.11° and −16.92° in longitude and between 0–2,355 m above sea level in altitude. Predicted δ^2^H_P_ from the sampling locations ranged between −32.8‰ and −3.4‰ according to [31]. Stable isotope ratios in fur keratin of pteropodids ranged from −160.0‰ to −47.5‰ for δ^2^H_K_, from −27.4‰ to −18.3‰ for δ^13^C, and from 3.9‰ to 18.3‰ for δ^15^N (Table 1). A MANOVA revealed significant differences in stable isotope ratios (δ^2^H_k_, δ^13^C, and δ^15^N) among locations (F_6,57_ = 23.4; p<0.001) in the two *Epomophorus* species. The discriminant function analysis showed that by using a triple-isotope approach, 84% of the *E. wahlbergi* and *E. crypturus* samples were assigned correctly to their location of capture (Fig. 1). Some locations formed isolated groups, e.g. those from city of Dar es Salaam and the Usambara and Lubumbashi region, while others presented a less clear separation in the plot, mostly those from the Democratic Republic of Congo (Fig. 1).

### The Predictive Power of Hydrogen Stable Isotope Ratios in Fur Keratin

**Figure 2:**
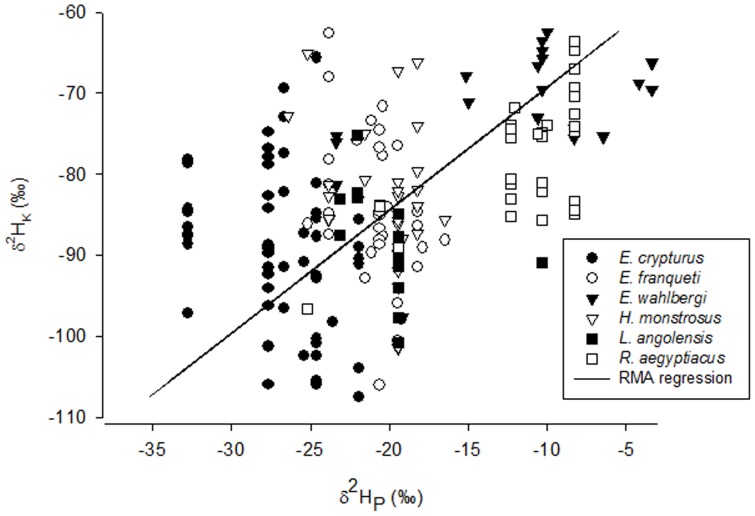
Relationship between δ^2^H_P_ and δ^2^H_K_ for non-migratory species (n = 193). δ^2^H_P_ was obtained from [**44**]. The solid line indicates the RMA regression.

δ^2^H_K_ and δ^2^H_P_ were significantly correlated in three out of the six non-migratory species. *E. wahlbergi* and *R. aegyptiacus* exhibited a positive correlation between δ^2^H_K_ and δ^2^H_P_ (Pearson’s *r* = 0.43 and 0.61; p = 0.038 and <0.001 respectively), while the four other species showed either no (*E. crypturus: r* = −0.23, p = 0.081; *L. angolensis*: *r* = −0.4, p = 0.107) or a negative correlation (*H. monstrosus*: *r* = −0.47; p = 0.007; and *E. franqueti*: *r* = −0.41; p = 0.023). The RMA regression calculated for data of all non-migratory species read: δ^2^H_P_ = 35.7+0.66 * δ^2^H_K_. The slope of the regression was significant (P<0.001; Fig. 2).

### Assessing the Migratory Behavior of *Eidolon helvum* Based on Hydrogen Stable Isotope Ratios

**Figure 3:**
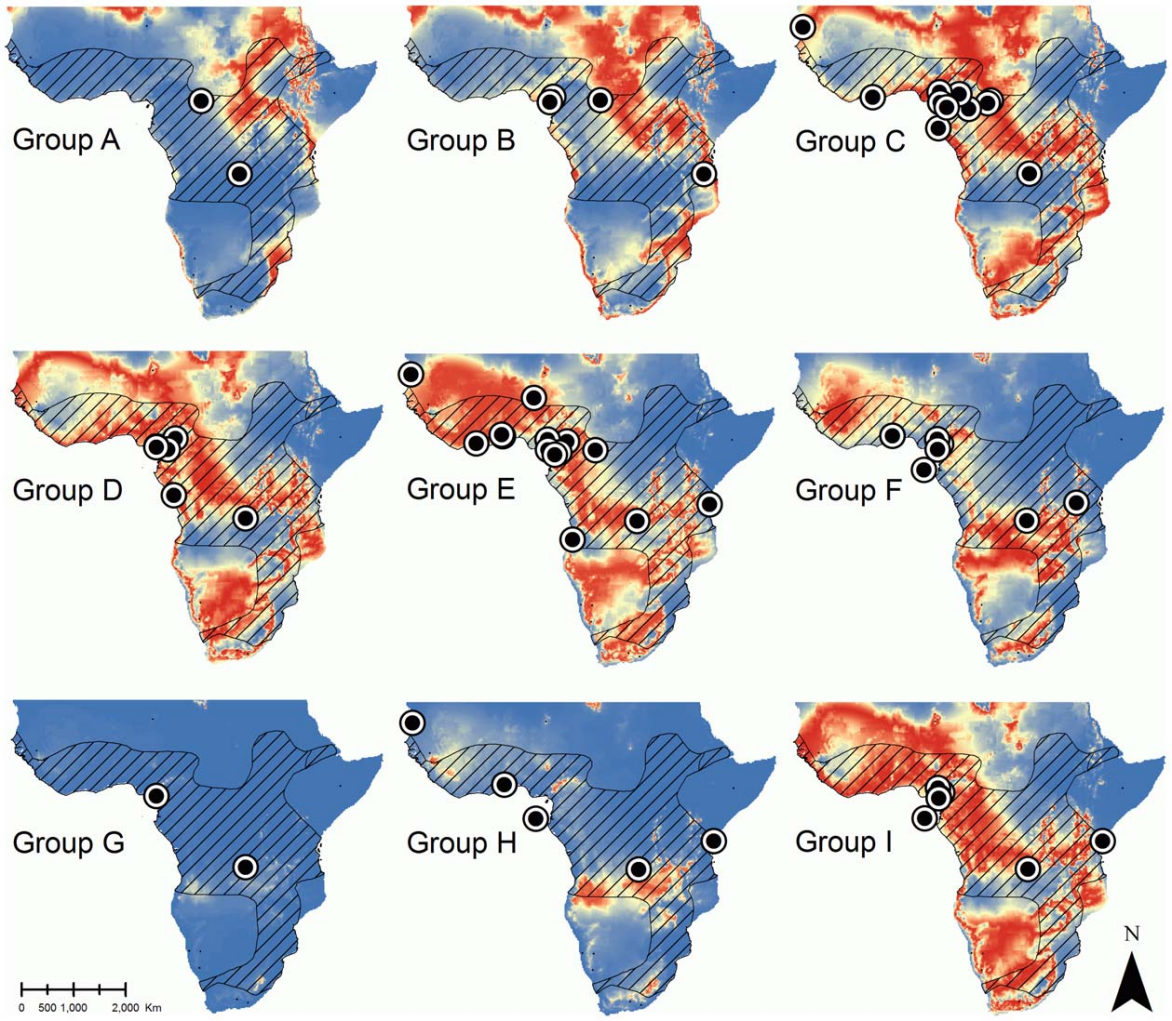
Mean probability maps for 88 individuals of *Eidolon helvum* (maps A - I). The IUCN species distribution range shown as an outline with diagonal lines. Based on our isoscape origin model, land areas are shaded on a scale from high (red) to low (blue) probability of origin, with intermediate probabilities indicated by pale yellow shading.

Based on the developed RMA regression equation and by taking error propagation into account, we transformed values of δ^2^H_K_ of *E. helvum* (n = 88) to create standardized probability density plots. We observed a large variance of δ^2^H_K_ in individuals of the same location, suggesting a variable migratory behavior in the study populations. We mapped nine mean probability maps for *E. helvum* (Fig. 3). In three cases, we obtained probability maps with a low probability of origin for the whole distribution of the species (map G for *E. helvum*). We were not able to assign a migratory distance to these three individuals. We categorized individuals with a minimum distance of ≤250 km between the capture location and the nearest most probable point of origin (threshold probability: 70%) as non-migratory, and individuals with a minimum distance of >250 km as migratory. In museum specimens of *E. helvum*, 77.6% (69 out of 85) of the individuals were non-migratory individuals with a median estimated minimum travel distance of 15 km (range: 0–250 km). We considered the remaining proportion (22.4%; 16 out of 85) as migratory, exhibiting a median minimum travel distance of about 860 km (range: 270–3,000 km). Some *E. helvum* individuals captured at the same locality and in the same campaign (month and/or year labeled at the museum) showed very different origin probabilities. For example, individuals captured in Nouakchott (Mauritania), Sao Tome and Principe, and Eidolon Land (Democratic Republic of Congo), which were classified in different mean probability groups (Fig. 3).

The stable isotope analysis of contemporary samples revealed that δ^2^H_k_ averaged −80.2±5.1‰ (minimum: −93.5‰; maximum: −62.4‰) for individuals from Dar es Salaam and −78.5±6.3 ‰ (minimum: −92.5‰; maximum: −70.4‰) for individuals from Morogoro. The subtle difference was not statistically significant (Student t-test: t_74_ = 1.19, P = 0.239). Nonetheless, we performed isotope origin model validation for the two sites separately, because δ^2^H_P_ differed between the two sites. These analyses showed a high discriminative power for both locations. The likelihood of *E. helvum* coming from the colony in Morogoro equaled 0.99 and for the Dar es Salaam colony 0.56. For the latter colony, the distance to the nearest spatial cluster with probability values higher than 70% was about 70 km.

## Discussion

We studied the migratory behavior of the straw-colored flying fox (*Eidolon helvum*) an important ecosystem service provider in sub-Saharan Africa, by means of stable isotope analysis and isoscape origin modeling. In our study, we used three steps that we will discuss under separate sub-headings.

### A Triple-isotope Approach to Geolocate Flying Foxes in Africa

We used a triple-isotope approach to evaluate whether the Sub-Saharan African isoscape was sufficiently heterogeneous to warrant the delineation of geographically distinct populations based on stable isotope ratios. We were able to distinguish among geographically distinct populations of *E. crypturus* and *E. wahlbergi* using stable isotopes of three elements (δ^2^H_K,_ δ^13^C_,_ and δ^15^N). The probability of assigning the geographic origin of a fur sample correctly averaged 84% for the seven localities analyzed, which we consider to be satisfactory given the overall low isotopic variation in large areas of the African continent; at least for stable isotopes of hydrogen [31], [34]. The discriminant function analysis showed a clear separation of most populations, even though the majority of samples were from a relatively small area in central Africa (Republic of Congo and Democratic Republic of Congo). Populations with a clear separation from others were those from the town of Dar es Salaam and the Usambara region (both Tanzania), and those collected from Lubumbashi (Democratic Republic of Congo). Samples coming from Lubumbashi were labeled as coming from the zoological garden. Fur keratin of these animals was ^15^N-enriched compared with fur keratin of most other pteropodids for which nitrogen isotope ratios are known [51], suggesting that they may have fed on agricultural plants that grew on fertilized soil. Possibly, they were also forming a captive population in the zoological garden and individuals were fed with fruits from some place distant from Lubumbashi or were facing nutritional stress. Nutritional stress is known to increase the variation or to cause systematic shifts in nitrogen isotope ratios [52]–[54]. In general, we assume that the spatial resolution of the multi-isotope approach for African flying foxes could be improved for African flying foxes by including more locations and also more elements in the analysis, as suggested for Afrotropical birds [34].

### The Predictive Power of Hydrogen Stable Isotope Ratios in Fur Keratin

Our general aim was to predict δ^2^H_P_ of the place of origin for the 88 *E. helvum* of the two museum collections based on δ^2^H_k_. To this end, we assessed whether δ^2^H_k_ is related to annual average δ^2^H_P_ of the location where six non-migratory species were captured. Overall, we found positive (*E. wahlbergi, R. aegyptiacus*), negative (*H. monstrosus* and *E. franqueti*) and lack of correlations (*E. crypturus, L. angolensis*). Likely explanations for the various direction of the relationships are (1) a biased concentration of samples, for example, from only one location or various neighboring locations, resulting in insufficient resolution to differentiate isotopic values between two localities (*E. crypturus, E. franqueti* and *H. monstrosus*), (2) low sample sizes per locality, with possible outlier effects (*L. angolensis*), (3) a high variance of isotopic data obtained from fur of a given capture site, possibly reflecting large variation of δ^2^H_P_ for a small area (*L. angolensis*), (4) using annual δ^2^H_P_ instead of monthly δ^2^H_P_ as the reference, and/or (5) simply because some species are not truly non-migratory but rather regional migratory species. For example, *E. helvum* did not show a correlation between δ^2^H_K_ and δ^2^H_P_, most likely because sampled *E. helvum* fur did not grow in the area where animals were captured. In addition, it is important to remember that large areas of the African continent are not surveyed for δ^2^H of precipitation water [30]. Therefore, predicted δ^2^H_P_ may not match the true values of precipitation water when extrapolated based on temperature, rainfall and altitude. Using annual δ^2^H_P_ instead of δ^2^H_P_ of the month when bats molt may be another important factor that may have obscured a positive correlation between δ^2^H_K_ and δ^2^H_P_. Seasonal variation in δ^2^H_P_ is large on the African continent [31], and therefore it is possible that we referred to a false value for δ^2^H_P_. Nonetheless, when analyzed together, we found a statistically significant correlation between δ^2^H_k_ and δ^2^H_P_ across all non-migratory species, even though intra-specific correlations of δ^2^H_k_ and δ^2^H_P_ did not show a consistent pattern, and even though we were not able to take the strong seasonal variation of δ^2^H_P_ in Sub-Saharan Africa into account.

### Assessing the Migratory Behavior of *Eidolon helvum* Based on Hydrogen Stable Isotope Ratios

For each *E. helvum*, we created predictive maps of origin using the established RMA and gamma distributions. This allowed the inaccuracy of measuring hydrogen isotopic ratios from fur samples and in predicting the hydrogen isotope ratios of local precipitation water to be taken into account. Our results showed that most *E. helvum* (78%; 69 out of 85) were captured from non-migratory populations. We estimated a median minimum travel distance of 15 km for the non-migratory animals, which is similar to what has been observed as daily commuting distances in *R. aegyptiacus* [7]. The ground-truthing of our isoscape origin model, based on stable isotope analysis in extant individuals of *E. helvum*, showed a high accuracy in predicting the origin of the inland colony in the city of Morogoro and a medium probability of origin for the individuals of the coastal site located in the city of Dar es Salaam. Possibly, the predicted δ^2^H_P_ of the coastal site is more error-prone than values from inland sites because of the high humidity and various influxes of water at coastal sites. Also, according to our estimate non-migratory *E. helvum* from the Dar es Salaam colony could have travelled for 70 km and reached δ^2^H_P_ that were similar to their δ^2^H_k_. The successful validation of our isoscape origin model for *E. helvum* is noteworthy because it was based on two large assumptions. Firstly, we referred to annual δ^2^H_P_ instead of monthly δ^2^H_K_ values and this should have increased the variance or added a systematic bias to our extrapolation. Secondly, we lacked information about the exact molting period of *E. helvum*. We assumed that molting would occur before a possible migration period. The successful validation of our isoscape origin model therefore supports the notion that these two assumptions were not largely violated in our stable isotope study of *E. helvum*.

We categorized 16 out of 85 *E. helvum* (22%) as migratory. The median minimum travel distance of migratory animals was 860 km, and four individuals seem to have traveled over a distance of more than 2,000 km prior to capture. This observation corroborates earlier findings that *E. helvum* is capable of performing long-distance migrations when following the seasonal fluctuation in fruit abundance [5]. Based on our literature review and the capture information from some museum specimens included in this study (only available for 7 localities), we assume that *E. helvum* has a core distribution in equatorial Africa, with migrations in northern direction, e.g. Mauritania, and Niger, from May to September and towards the south, e.g. Tanzania, Zimbabwe and Zambia during the months of October and December. Individuals captured at the same time and at the same locality in Central Africa exhibited contrasting isotopic signatures in their fur, and in consequence are likely to have originated from different locations. For example, this pattern was apparent in *E. helvum* individuals captured in Nouakchott (Mauritania; n = 4), where two individuals presented an isotopic origin from the same locality, yet two individuals originated from localities at around 250 km and 850 km away from the collection site. Individuals captured at Eidolon land, near the National Park of Kundelungu (Democratic Republic of Congo; n = 10) also showed this pattern, with eight non-migratory individuals, and two having migrated over a distance of about 700 km.

For three individuals, we obtained a mean probability map with a low probability of origin for the whole continent (*E. helvum* group G). This was caused by low δ^2^H_k_ values that were not typical for the average annual δ^2^H_P_ of the African continent, i.e. once transformed with the RMA and simulated with the gamma function, these values remained outside the data range of δ^2^H_P_ of Africa. One possible explanation is that our isoscape origin model was based on annual mean δ^2^H_P_ of Africa [45], and these individuals came from regions where stable hydrogen isotope ratios vary largely during a short period of time, as it is the case for example in West Africa in August (www.waterisotopes.org). Consequently, these bats may have carried a stable hydrogen isotope signature that was representative for a small area or a brief period of time for the specific capture location. Alternatively, these specimens may have been maintained in captivity and fed with non-local fruits between collection and shipment to the museum collections. This raises an important point about using museum specimens in stable isotope studies: circumstances of capture and the exact Julian date of capture are usually not documented in museum specimens. Also, it is not usually documented how specimens were treated after sacrificing and shipping them to the museums, e.g. by using different preservatives at the site of collection, during shipment and for permanent storage in the museums.

### Museum Collections and Stable Isotope Studies

Specimens from museum collections are increasingly used to study the isotopic ecology of vertebrates. In our study, the use of specimens from museum collection was constrained, because of the lack of information about the Julian date of capture or the change in the naming of locations over the past century. Also, it was unknown for all studied museum specimens whether bats were captured in their natural habitat or, for example, purchased from local markets. Our isotopic origin model would have suffered if bats collected for museums originated from other locations than indicated by the collector. However, since it is unlikely that wrong location assignments caused a systematic bias, we are confident that the general picture of our study remains unaffected by this possibility. In addition, ethanol preserving could have increased the variance in stable isotope ratios in museum specimens, if ethanol interacts with the keratinous protein. Yet, we found a positive correlation between δ^2^H_k_ and δ^2^H_P_ in non-migratory species, suggesting that effects of preservatives were probably absent or negligible. In summary, museum collections allowed us to obtain a high number of samples at a low cost and to provide new data about the movement ecology of an ecological and economical key species on the African continent.

### Conclusions

Our study is one of the first to show that stable isotope ratios can be used to predict the provenance of animals on the African continent. One recent study in a long-distant bird migrant carrying geolocators confirmed that feather δ^2^H_K_ of northern wheatear, *Oenanthe oenanthe*, recorded the variation of the predicted δ^2^H_P_ of the overwintering sites [55]. Other studies have also supported a large potential of using stable isotope ratios for investigating the movement ecology of Afro-European birds [34], [56]. Yet except for these and some other papers, stable isotope studies on migratory African birds or bats have been rare or absent respectively. The major reason for this is that the isoscape of the African continent has been considered to be less informative than those of, for example, Europe and North America [31]. Our study shows that a triple-isotope approach (δ^2^H_k,_ δ^13^C and δ^15^N) is useful to discriminate between fur samples of flying foxes that originated from various locations in Central Africa. However, since this approach requires a complete baseline map for the stable isotope ratios in keratin of non-migratory species, it may not be feasible on a larger geographic scale. Therefore, we have validated that δ^2^H_K_ is correlated positively with δ^2^H_P_ across individuals of non-migratory flying foxes. Based on the developed RMA regression and the consideration of error propagation, we used an isoscape origin model to estimate the movement ecology of *E. helvum*. Using historical specimens of this species, we confirmed that a large portion of the population was non-migratory and that some individuals performed long-distance migrations of more than 2,000 km; a finding that corroborates earlier studies using satellite tracking [18]. Overall, we observed travel distances of only about 870 km in migratory individuals. This could be attributed to the observation that *E. helvum* may perform several small-scale migrations of a few 100 km from one stepping stone to the other, following the seasonal abundance of local food resources on the Sub-Saharan African continent.

## Supporting Information

Figure S1 Location of collection sites for each of the seven flying fox species. The African continent is coloured in relation the annual average stable hydrogen isotope ratio of precipitation water according to [31]. The distribution range of flying foxes is marked as a shaded area.
(TIF)

Table S1 Reference numbers of the sampled specimens at the National Museum for Natural History in Paris (MNHN) and the Natural History Museum in Berlin (MFN) and the corresponding stable isotope ratios for carbon (δ^13^C), nitrogen (δ^15^N) and hydrogen (δ^2^H_K_). Abbreviations for countries are: ZIM = Zimbabwe. DRC = Democratic Republic of Congo. GHA = Ghana. CAM = Cameroon. NIG = Nigeria. ANG = Angola. MAU = Mauritania. TAN = Tanzania. SAF = South Africa. STP = Sao Tome and Principe. IVO = Ivory coast. CAR = Central African Republic. RC = Republic of Congo (na = not available, Alt. = altitude, Lat. = latitude, Long. = longitude).
(DOCX)
