# Supplementary material for: The Movement Ecology of the Straw-Colored Fruit Bat, Eidolon helvum, in Sub-Saharan Africa Assessed by Stable Isotope Ratios
Source: PLoS One. 2012 Sep 21;7(9):e45729. doi: 10.1371/journal.pone.0045729 (PMC3448674; doi:10.1371/journal.pone.0045729)
Supplement: Table S1 — Reference numbers of the sampled specimens at the National Museum for Natural History in Paris (MNHN) and the Natural History Museum in Berlin (MFN) and the corresponding stable isotope ratios for carbon (δ13C), nitrogen (δ15N) and hydrogen (δ2HK). Abbreviations for countries are: ZIM = Zimbabwe. DRC = Democratic Republic of Congo. GHA = Ghana. CAM = Cameroon. NIG = Nigeria. ANG = Angola. MAU = Mauritania. TAN = Tanzania. SAF = South Africa. STP = Sao Tome and Principe. IVO = Ivory coast. CAR = Central African Republic. RC = Republic of Congo (na = not available, Alt. = altitude, Lat. = latitude, Long. = longitude). (DOCX) [file pone.0045729.s002.docx]

**Electronic supplementary material Table S1**

| **Number** | **Museum** | **Year** | **Country** | **Location** | **Alt.** | **Lat.** | **Long.** | **δ^13^C** | **δ^15^N** | **δ^2^H_K_** |
| --- | --- | --- | --- | --- | --- | --- | --- | --- | --- | --- |
| **EC** |  |  |  |  |  |  |  |  |  |  |
| 1990-135 | MNHN | na | ZIM | Kudzwe River | 724 | -17.05 | 32.17 | -21.5 | 8.0 | -82.1 |
| 1990-138 | MNHN | na | ZIM | Kudzwe River | 724 | -17.05 | 32.17 | -21.1 | 8.7 | -69.3 |
| 1990-139 | MNHN | na | ZIM | Kudzwe River | 724 | -17.05 | 32.17 | -22.3 | 6.8 | -96.5 |
| 1990-141 | MNHN | na | ZIM | Kudzwe River | 724 | -17.05 | 32.17 | -21.1 | 6.9 | -77.4 |
| 1990-147 | MNHN | na | ZIM | Kudzwe River | 724 | -17.05 | 32.17 | -21.2 | 7.1 | -73.0 |
| 1990-148 | MNHN | na | ZIM | Kudzwe River | 724 | -17.05 | 32.17 | -21.8 | 6.9 | -91.4 |
| 2011-727 | MNHN | 2009 | DRC | Forzt | 1155 | -10.12 | 26.74 | -22.1 | 5.3 | -89.7 |
| 2011-728 | MNHN | 2009 | DRC | Forzt | 1155 | -10.12 | 26.74 | -22.1 | 4.8 | -91.5 |
| 2011-729 | MNHN | 2009 | DRC | Forzt | 1155 | -10.12 | 26.74 | -21.7 | 4.3 | -77.9 |
| 2011-730 | MNHN | 2009 | DRC | Forzt | 1155 | -10.12 | 26.74 | -22.6 | 5.8 | -82.7 |
| 2011-731 | MNHN | 2009 | DRC | Forzt | 1155 | -10.12 | 26.74 | -22.2 | 3.9 | -89.1 |
| 2011-732 | MNHN | 2009 | DRC | Forzt | 1155 | -10.12 | 26.74 | -22.3 | 5.3 | -84.2 |
| 2011-733 | MNHN | 2009 | DRC | Forzt | 1155 | -10.12 | 26.74 | -22.1 | 4.9 | -101.2 |
| 2011-734 | MNHN | 2009 | DRC | Forzt | 1155 | -10.12 | 26.74 | -21.6 | 5.9 | -78.8 |
| 2011-735 | MNHN | 2009 | DRC | Forzt | 1155 | -10.12 | 26.74 | -21.1 | 4.7 | -76.8 |
| 2011-736 | MNHN | 2009 | DRC | Forzt | 1155 | -10.12 | 26.74 | -22.9 | 5.1 | -94.1 |
| 2011-737 | MNHN | 2009 | DRC | Forzt | 1155 | -10.12 | 26.74 | -22.1 | 6.3 | -96.2 |
| 2011-738 | MNHN | 2009 | DRC | Forzt | 1155 | -10.12 | 26.74 | -22.2 | 5.0 | -74.8 |
| 2011-739 | MNHN | 2009 | DRC | Forzt | 1155 | -10.12 | 26.74 | -22.7 | 5.0 | -105.9 |
| 2011-740 | MNHN | 2009 | DRC | Forzt | 1155 | -10.12 | 26.74 | -22.4 | 5.3 | -88.7 |
| 2011-742 | MNHN | 2009 | DRC | Forzt | 1155 | -10.12 | 26.74 | -22.2 | 5.3 | -92.4 |
| 2011-743 | MNHN | 2009 | DRC | Kaloba | 1719 | -8.23 | 25.70 | -22.4 | 6.0 | -100.8 |
| 2011-744 | MNHN | 2009 | DRC | Kaloba | 1719 | -8.23 | 25.70 | -22.2 | 5.1 | -105.9 |
| 2011-745 | MNHN | 2009 | DRC | Kaloba | 1719 | -8.23 | 25.70 | -22.4 | 6.0 | -84.8 |
| 2011-746 | MNHN | 2009 | DRC | Kaloba | 1719 | -8.23 | 25.70 | -22.0 | 5.8 | -102.4 |
| 2011-747 | MNHN | 2009 | DRC | Kaloba | 1719 | -8.23 | 25.70 | -22.8 | 5.4 | -100.3 |
| 2011-748 | MNHN | 2009 | DRC | Kaloba | 1719 | -8.23 | 25.70 | -21.9 | 5.7 | -65.6 |
| 2011-749 | MNHN | 2009 | DRC | Kaloba | 1719 | -8.23 | 25.70 | -21.6 | 5.2 | -81.2 |
| 2011-750 | MNHN | 2009 | DRC | Kaloba | 1719 | -8.23 | 25.70 | -21.5 | 5.2 | -92.5 |
| 2011-751 | MNHN | 2009 | DRC | Kaloba | 1719 | -8.23 | 25.70 | -22.1 | 5.1 | -92.8 |
| 2011-752 | MNHN | 2009 | DRC | Kaloba | 1719 | -8.23 | 25.70 | -22.4 | 5.2 | -105.4 |
| 2011-753 | MNHN | 2009 | DRC | Kaloba | 1719 | -8.23 | 25.70 | -22.2 | 5.3 | -105.9 |
| 2011-754 | MNHN | 2009 | DRC | Kaloba | 1719 | -8.23 | 25.70 | -21.8 | 4.3 | -85.3 |
| 2011-755 | MNHN | 2009 | DRC | Kaloba | 1719 | -8.23 | 25.70 | -22.4 | 5.1 | -87.6 |
| 2011-756 | MNHN | 2009 | DRC | Kasombo | 1318 | -5.31 | 18.87 | -21.9 | 6.3 | -97.9 |
| 2011-759 | MNHN | 2009 | DRC | Kimbongo | 612 | -6.13 | 18.01 | -22.7 | 6.4 | -85.5 |
| 2011-760 | MNHN | 2009 | DRC | Kimbongo | 612 | -6.13 | 18.01 | -23.5 | 6.4 | -88.9 |
| 2011-761 | MNHN | 2009 | DRC | Kimbongo | 612 | -6.13 | 18.01 | -22.3 | 5.9 | -91.0 |
| 2011-762 | MNHN | 2009 | DRC | Kimbongo | 612 | -6.13 | 18.01 | -21.9 | 7.6 | -82.9 |
| 2011-763 | MNHN | 2009 | DRC | Kimbongo | 612 | -6.13 | 18.01 | -23.1 | 6.7 | -90.4 |
| 2011-764 | MNHN | 2009 | DRC | Kimbongo | 612 | -6.13 | 18.01 | -23.5 | 6.3 | -107.4 |
| 2011-765 | MNHN | 2009 | DRC | Kimbongo | 612 | -6.13 | 18.01 | -23.4 | 7.4 | -103.9 |
| 2011-766 | MNHN | 2009 | DRC | Lubumbashi | 1209 | -11.66 | 27.48 | -22.6 | 13.6 | -78.6 |
| 2011-767 | MNHN | 2009 | DRC | Lubumbashi | 1209 | -11.66 | 27.48 | -23.1 | 12.5 | -84.7 |
| 2011-768 | MNHN | 2009 | DRC | Lubumbashi | 1209 | -11.66 | 27.48 | -22.7 | 12.4 | -86.5 |
| 2011-769 | MNHN | 2009 | DRC | Lubumbashi | 1209 | -11.66 | 27.48 | -23.0 | 11.6 | -78.2 |
| 2011-770 | MNHN | 2009 | DRC | Lubumbashi | 1209 | -11.66 | 27.48 | -23.0 | 11.5 | -88.6 |
| 2011-771 | MNHN | 2009 | DRC | Lubumbashi | 1209 | -11.66 | 27.48 | -22.7 | 12.8 | -87.6 |
| 2011-772 | MNHN | 2009 | DRC | Lubumbashi | 1209 | -11.66 | 27.48 | -22.5 | 12.6 | -87.4 |
| 2011-773 | MNHN | 2009 | DRC | Lubumbashi | 1209 | -11.66 | 27.48 | -22.2 | 11.9 | -97.1 |
| 2011-774 | MNHN | 2009 | DRC | Lubumbashi | 1209 | -11.66 | 27.48 | -21.8 | 7.5 | -84.2 |
| 2011-775 | MNHN | 2009 | DRC | Mukulakulu | 796 | -9.64 | 25.83 | -23.0 | 7.6 | -102.3 |
| 2011-776 | MNHN | 2009 | DRC | Mukulakulu | 796 | -9.64 | 25.83 | -22.8 | 6.2 | -87.3 |
| 2011-777 | MNHN | 2009 | DRC | Mukulakulu | 796 | -9.64 | 25.83 | -22.5 | 7.8 | -90.8 |
| 2011-778 | MNHN | 2009 | DRC | Muvule | 1093 | -9.19 | 27.21 | -22.1 | 5.4 | -89.2 |
| 2011-779 | MNHN | 2009 | DRC | Muvule | 1093 | -9.19 | 27.21 | -21.8 | 4.6 | -88.8 |
| 2011-780 | MNHN | 2009 | DRC | PN Upemba | 842 | -8.91 | 26.58 | -23.2 | 7.0 | -98.2 |
| **EF** |  |  |  |  |  |  |  |  |  |  |
| 3562 | MFN | na | GHA | Ghana | 125 | 8.21 | -1.11 | -22.0 | 11.4 | -73.5 |
| 3649 | MFN | na | NIG | Lagos | 10 | 6.45 | 3.40 | -22.3 | 6.8 | -89.0 |
| 4785 | MFN | 1920 | CAM | Cameroon | 710 | 5.58 | 12.71 | -22.1 | 6.9 | -87.4 |
| 4956 | MFN | na | Gabon | Donguila | 58 | 0.21 | 9.74 | -21.4 | 6.0 | -88.1 |
| 5002 | MFN | na | CAM | Cameroon | 710 | 5.58 | 12.71 | -22.0 | 9.1 | -84.8 |
| 8945 | MFN | na | GHA | Kete | 117 | 7.83 | -0.05 | -22.3 | 6.1 | -87.5 |
| 8946 | MFN | na | GHA | Kete | 117 | 7.83 | -0.05 | -22.3 | 8.2 | -77.7 |
| 9041 | MFN | na | CAM | Bipindi | 78 | 3.08 | 10.41 | -22.6 | 6.8 | -84.7 |
| 9972 | MFN | na | CAM | Cameroon | 710 | 5.58 | 12.71 | -21.0 | 12.5 | -68.0 |
| 9973 | MFN | na | CAM | Cameroon | 710 | 5.58 | 12.71 | -19.4 | 10.3 | -62.6 |
| 10004 | MFN | na | Togo | Togo | 223 | 8.00 | 1.16 | -21.7 | 8.4 | -75.8 |
| 10031 | MFN | na | DRC | Malandji | 562 | -5.92 | 22.28 | -22.3 | 6.8 | -84.0 |
| 40160 | MFN | na | CAM | Bipindi | 78 | 3.08 | 10.41 | -23.5 | 10.2 | -88.1 |
| 40161 | MFN | na | CAM | Bipindi | 78 | 3.08 | 10.41 | -22.6 | 7.9 | -85.1 |
| 50003 | MFN | 1904 | Togo | Misahöhe | 295 | 6.93 | 0.60 | -24.0 | 5.7 | -92.8 |
| 53871 | MFN | 1898 | CAM | Limbe | 36 | 4.02 | 9.20 | -23.7 | 7.4 | -95.9 |
| 53872 | MFN | 1902 | CAM | Limbe | 36 | 4.02 | 9.20 | -27.4 | 7.2 | -100.5 |
| 53874 | MFN | 1899 | GHA | Kete | 117 | 7.80 | -0.05 | -19.8 | 4.0 | -71.7 |
| 53916 | MFN | 1899 | CAM | Cameroon | 710 | 5.58 | 12.71 | -22.9 | 7.6 | -81.2 |
| 54198 | MFN | na | CAM | Bipindi | 78 | 3.08 | 10.41 | -22.8 | 9.5 | -88.6 |
| 54520 | MFN | na | CAM | Yaounde | 726 | 3.87 | 11.52 | -22.3 | 7.4 | -86.1 |
| 54933 | MFN | 1904 | CAM | Longji | 23 | 3.08 | 9.97 | -22.9 | 8.5 | -91.4 |
| 54934 | MFN | 1898 | CAM | Bipindi | 78 | 3.08 | 10.41 | -23.1 | 9.3 | -86.7 |
| 67052 | MFN | 1899 | CAM | Limbe | 36 | 4.02 | 9.20 | -22.6 | 7.8 | -76.4 |
| 67098 | MFN | 1912 | CAM | Bipindi | 78 | 3.08 | 10.41 | -22.5 | 8.5 | -84.7 |
| 67101 | MFN | 1907 | CAM | Bipindi | 78 | 3.08 | 10.41 | -22.8 | 9.2 | -74.6 |
| 67102 | MFN | 1908 | CAM | Cameroon | 710 | 5.58 | 12.71 | -21.6 | 8.0 | -78.2 |
| 67103 | MFN | 1909 | CAM | Batonga | 28 | 2.84 | 9.89 | -22.7 | 5.4 | -86.4 |
| 67104 | MFN | 1910 | CAM | Bipindi | 78 | 3.08 | 10.41 | -23.7 | 7.8 | -105.9 |
| 67105 | MFN | 1907 | CAM | Bipindi | 78 | 3.08 | 10.41 | -22.8 | 9.8 | -76.7 |
| **EH** |  |  |  |  |  |  |  |  |  |  |
| 4239 | MFN | na | ANG | Benguela | 424 | -12.73 | 13.64 | -22.0 | 7.1 | -91.3 |
| 4951 | MFN | na | Gabon | Cap Lopez | 0 | -0.77 | 8.84 | -21.7 | 7.2 | -94.7 |
| 5022 | MFN | 1874 | CAM | Cameroon | 710 | 5.58 | 12.71 | -20.4 | 10.4 | -89.8 |
| 5224 | MFN | na | ANG | Cabinda | 95 | -5.02 | 12.35 | -21.2 | 8.1 | -83.2 |
| 10203 | MFN | 1891 | CAM | Buea | 963 | 4.16 | 9.23 | -21.4 | 8.6 | -82.1 |
| 10204 | MFN | na | CAM | Buea | 963 | 4.16 | 9.23 | -21.6 | 8.2 | -88.6 |
| 54318 | MFN | 1898 | CAM | Cameroon | 710 | 5.58 | 12.71 | -21.8 | 11.0 | -83.6 |
| 54523 | MFN | na | CAM | Limbe | 36 | 4.02 | 9.20 | -20.8 | 8.3 | -69.6 |
| 54524 | MFN | 1902 | CAM | Limbe | 36 | 4.02 | 9.20 | -20.7 | 8.2 | -82.6 |
| 54597 | MFN | 1899 | CAM | Limbe | 36 | 4.02 | 9.20 | -21.2 | 7.3 | -76.0 |
| 54598 | MFN | 1905 | CAM | Ossidinge | 1974 | 5.91 | 9.11 | -21.0 | 6.9 | -95.0 |
| 54602 | MFN | 1905 | CAM | Ossidinge | 1974 | 5.91 | 9.11 | -21.4 | 7.9 | -95.6 |
| 54603 | MFN | 1901 | TAN | Tanzania | 829 | -6.00 | 35.00 | -21.2 | 7.1 | -98.0 |
| 54674 | MFN | 1938 | CAM | Victoria | 24 | 4.01 | 9.22 | -21.8 | 6.9 | -160.0 |
| 54692 | MFN | 1899 | CAM | Limbe | 36 | 4.02 | 9.20 | -20.0 | 7.8 | -84.2 |
| 54693 | MFN | 1905 | CAM | Ossidinge | 1974 | 5.91 | 9.11 | -21.5 | 8.7 | -81.0 |
| 54701 | MFN | 1901 | TAN | Tanzania | 829 | -6.00 | 35.00 | -20.6 | 5.5 | -96.4 |
| 54702 | MFN | 1905 | CAM | Ossidinge | 1974 | 5.91 | 9.11 | -19.9 | 8.1 | -77.5 |
| 54703 | MFN | 1901 | CAM | Moba | 584 | 2.78 | 14.50 | -21.7 | 8.6 | -79.6 |
| 54935 | MFN | 1905 | CAM | Ossidinge | 1974 | 5.91 | 9.11 | -21.2 | 7.8 | -86.1 |
| 54939 | MFN | 1905 | CAM | Ossidinge | 1974 | 5.91 | 9.11 | -21.5 | 7.2 | -93.7 |
| 54940 | MFN | 1899 | CAM | Limbe | 36 | 4.02 | 9.20 | -21.2 | 7.8 | -90.9 |
| 54941 | MFN | 1899 | TAN | Ruhangino Lindi | 585 | -9.43 | 37.87 | -20.1 | 6.9 | -70.4 |
| 58209 | MFN | na | TAN | Zanzibar | 33 | -6.17 | 39.20 | -20.4 | 9.5 | -88.0 |
| 67045 | MFN | 1903 | CAM | Cameroon | 710 | 5.58 | 12.71 | -20.8 | 8.6 | -77.8 |
| 67046 | MFN | 1938 | CAM | Victoria | 24 | 4.01 | 9.22 | -22.5 | 6.9 | -96.6 |
| 67136 | MFN | 1899 | CAM | Limbe | 36 | 4.02 | 9.20 | -20.6 | 8.3 | -79.9 |
| 67140 | MFN | 1902 | CAM | Limbe | 36 | 4.02 | 9.20 | -21.0 | 8.2 | -80.8 |
| 67141 | MFN | 1902 | CAM | Limbe | 36 | 4.02 | 9.20 | -22.0 | 8.2 | -85.9 |
| 67142 | MFN | 1906 | CAM | Bipindi | 78 | 3.08 | 10.41 | -19.5 | 9.1 | -78.3 |
| 67143 | MFN | 1906 | CAM | Bipindi | 78 | 3.08 | 10.41 | -20.0 | 8.6 | -93.0 |
| 67144 | MFN | 1938 | CAM | Victoria | 24 | 4.01 | 9.22 | -20.5 | 8.0 | -85.2 |
| 67145 | MFN | 1938 | CAM | Victoria | 24 | 4.01 | 9.22 | -21.7 | 6.0 | -126.7 |
| 1881-290 | MNHN | na | Kenya | Mombassa | 1 | -4.04 | 39.67 | -21.2 | 10.2 | -111.7 |
| 1881-291 | MNHN | na | Kenya | Mombassa | 1 | -4.04 | 39.67 | -20.4 | 7.0 | -85.8 |
| 1911-778 | MNHN | na | Niger | Zamia | 299 | 13.61 | 6.63 | -21.0 | 7.6 | -87.6 |
| 1975-776 | MNHN | na | Togo | Palime | 246 | 6.90 | 0.63 | -21.8 | 6.8 | -89.1 |
| 1975-777 | MNHN | 1971 | SAF | South Africa | 137 | 0.23 | 6.62 | -22.8 | 7.1 | -96.2 |
| 1975-778 | MNHN | 1971 | SAF | South Africa | 137 | 0.23 | 6.62 | -22.2 | 6.8 | -84.1 |
| 1975-779 | MNHN | 1971 | SAF | South Africa | 137 | 0.23 | 6.62 | -22.1 | 7.9 | -108.7 |
| 1975-783 | MNHN | na | CAR | La Maboke | 515 | 3.87 | 17.99 | -23.1 | 10.6 | -91.2 |
| 1975-784 | MNHN | 1963 | Togo | Kouda Topki | 246 | 6.63 | 0.86 | -22.3 | 6.6 | -99.7 |
| 1975-807 | MNHN | 1963 | Togo | Kouda Topki | 246 | 6.63 | 0.86 | -22.6 | 9.2 | -90.9 |
| 1975-814 | MNHN | na | IVO | Assinie | 0 | 5.14 | -3.32 | -21.2 | 10.2 | -80.1 |
| 1975-817 | MNHN | 1962 | CAR | La Maboke | 515 | 3.87 | 17.99 | -23.0 | 9.7 | -80.7 |
| 1975-820 | MNHN | 1963 | Togo | Kouda Topki | 246 | 6.63 | 0.86 | -22.7 | 9.1 | -107.3 |
| 1975-821 | MNHN | 1963 | Togo | Kouda Topki | 246 | 6.63 | 0.86 | -22.9 | 8.5 | -93.8 |
| 1975-822 | MNHN | 1963 | Togo | Kouda Topki | 246 | 6.63 | 0.86 | -22.6 | 8.3 | -91.6 |
| 1975-823 | MNHN | 1963 | Togo | Kouda Topki | 246 | 6.63 | 0.86 | -22.6 | 7.9 | -96.9 |
| 1975-824 | MNHN | 1963 | Togo | Kouda Topki | 246 | 6.63 | 0.86 | -22.5 | 7.6 | -107.2 |
| 1975-825 | MNHN | 1963 | Togo | Kouda Topki | 246 | 6.63 | 0.86 | -22.4 | 9.7 | -106.5 |
| 1975-826 | MNHN | 1963 | Togo | Kouda Topki | 246 | 6.63 | 0.86 | -23.2 | 7.5 | -102.6 |
| 1975-827 | MNHN | 1963 | Togo | Kouda Topki | 246 | 6.63 | 0.86 | -23.0 | 9.2 | -92.7 |
| 1975-830 | MNHN | 1963 | Togo | Kouda Topki | 246 | 6.63 | 0.86 | -23.2 | 7.9 | -94.1 |
| 1975-831 | MNHN | 1963 | Togo | Kouda Topki | 246 | 6.63 | 0.86 | -22.5 | 8.7 | -100.7 |
| 1975-856 | MNHN | na | IVO | Adiopodoume | 15 | 5.34 | -4.13 | -21.8 | 8.6 | -88.1 |
| 1979-314 | MNHN | 1973 | CAM | Mt Manengouba | 1559 | 5.03 | 9.83 | -21.3 | 7.8 | -89.0 |
| 1979-315 | MNHN | 1973 | CAM | Mt Manengouba | 1559 | 5.03 | 9.83 | -21.4 | 7.6 | -78.8 |
| 1979-316 | MNHN | 1973 | CAM | Mt Manengouba | 1559 | 5.03 | 9.83 | -22.0 | 6.2 | -86.0 |
| 1979-317 | MNHN | 1973 | CAM | Mt Manengouba | 1559 | 5.03 | 9.83 | -21.0 | 8.2 | -78.6 |
| 1979-318 | MNHN | 1973 | CAM | Mt Manengouba | 1559 | 5.03 | 9.83 | -20.7 | 8.7 | -73.0 |
| 1979-319 | MNHN | 1973 | CAM | Mt Manengouba | 1559 | 5.03 | 9.83 | -20.9 | 7.9 | -76.2 |
| 1979-320 | MNHN | 1973 | CAM | Mt Manengouba | 1559 | 5.03 | 9.83 | -22.5 | 9.4 | -98.0 |
| 1979-321 | MNHN | 1973 | CAM | Mt Manengouba | 1559 | 5.03 | 9.83 | -22.3 | 8.8 | -87.6 |
| 1979-322 | MNHN | 1973 | CAM | CAM | 710 | 5.58 | 12.71 | -22.0 | 9.5 | -77.1 |
| 1984-252 | MNHN | na | CAR | La Maboke | 515 | 3.87 | 17.99 | -23.7 | 9.9 | -93.1 |
| 1984-253 | MNHN | na | CAR | La Maboke | 515 | 3.87 | 17.99 | -24.5 | 10.2 | -77.3 |
| 1997-2105 | MNHN | 1995 | MAU | Nouakchott | 3 | 18.08 | -15.98 | -22.2 | 9.3 | -77.4 |
| 1997-2106 | MNHN | 1995 | MAU | Nouakchott | 3 | 18.08 | -15.98 | -22.4 | 7.1 | -75.6 |
| 1997-2107 | MNHN | 1995 | MAU | Nouakchott | 3 | 18.08 | -15.98 | -21.9 | 8.8 | -87.0 |
| 1997-2108 | MNHN | 1995 | MAU | Nouakchott | 3 | 18.08 | -15.98 | -23.4 | 7.5 | -112.3 |
| 2008- 97 | MNHN | na | CAR | Bangui | 369 | 4.36 | 18.56 | -22.4 | 7.8 | -74.2 |
| 2008- 98 | MNHN | na | CAR | Bangui | 369 | 4.36 | 18.56 | -22.5 | 6.2 | -78.9 |
| 2008- 99 | MNHN | na | CAR | Bangui | 369 | 4.36 | 18.56 | -22.6 | 4.9 | -65.5 |
| 2011- 638 | MNHN | 2007 | CAM | PN Campo Maan | 453 | 3.53 | 11.31 | -22.2 | 9.3 | -91.3 |
| 2011- 639 | MNHN | 2007 | CAM | PN Campo Maan | 453 | 3.53 | 11.31 | -22.0 | 7.3 | -83.6 |
| 2011- 684 | MNHN | 2008 | CAR | Bangui | 369 | 4.36 | 18.56 | -22.0 | 8.1 | -80.4 |
| 2011- 715 | MNHN | 2009 | DRC | Eidolon land | 1712 | -9.34 | 25.72 | -22.4 | 7.1 | -94.2 |
| 2011- 716 | MNHN | 2009 | DRC | Eidolon land | 1712 | -9.34 | 25.72 | -23.5 | 6.8 | -119.1 |
| 2011- 717 | MNHN | 2009 | DRC | Eidolon land | 1712 | -9.34 | 25.72 | -23.5 | 6.5 | -92.4 |
| 2011- 718 | MNHN | 2009 | DRC | Eidolon land | 1712 | -9.34 | 25.72 | -22.3 | 8.3 | -61.7 |
| 2011- 719 | MNHN | 2009 | DRC | Eidolon land | 1712 | -9.34 | 25.72 | -22.5 | 6.6 | -80.1 |
| 2011- 720 | MNHN | 2009 | DRC | Eidolon land | 1712 | -9.34 | 25.72 | -22.4 | 8.2 | -61.6 |
| 2011- 721 | MNHN | 2009 | DRC | Eidolon land | 1712 | -9.34 | 25.72 | -23.7 | 7.9 | -87.6 |
| 2011- 722 | MNHN | 2009 | DRC | Eidolon land | 1712 | -9.34 | 25.72 | -23.0 | 6.8 | -103.4 |
| 2011- 723 | MNHN | 2009 | DRC | Eidolon land | 1712 | -9.34 | 25.72 | -21.9 | 8.1 | -88.1 |
| 2011- 724 | MNHN | 2009 | DRC | Eidolon land | 1712 | -9.34 | 25.72 | -22.9 | 8.8 | -82.9 |
| 2011- 725 | MNHN | 2009 | DRC | Eidolon land | 1712 | -9.34 | 25.72 | -23.9 | 5.9 | -97.4 |
| **EW** |  |  |  |  |  |  |  |  |  |  |
| 4240 | MFN | na | ANG | Benguela | 424 | -12.73 | 13.64 | -18.3 | 18.3 | -72.5 |
| 4786 | MFN | na | GHA | Accra | 32 | 5.56 | -0.20 | -22.8 | 8.0 | -97.6 |
| 5081 | MFN | na | Kenya | Mombassa | 1 | -4.04 | 39.67 | -20.8 | 6.3 | -68.8 |
| 10014 | MFN | na | SAF | Grahams- town | 548 | -33.31 | 26.52 | -20.3 | 9.5 | -67.8 |
| 10016 | MFN | na | SAF | Kwazulu. Natal. Durban | 17 | -29.86 | 31.03 | -20.5 | 9.5 | -75.3 |
| 53898 | MFN | na | Kenya | Kibwezi | 899 | -2.41 | 37.97 | -20.2 | 10.6 | -62.4 |
| 54196 | MFN | 1912 | TAN | Tanzania | 829 | -6.00 | 35.00 | -20.6 | 7.7 | -71.2 |
| 54208 | MFN | na | TAN | Dar es Salaam | 21 | -6.82 | 39.27 | -19.7 | 7.2 | -73.0 |
| 54364 | MFN | 1901 | Kenya | Takaungu | 3 | -3.68 | 39.86 | -20.3 | 9.0 | -69.5 |
| 54366 | MFN | 1901 | Kenya | Takaungu | 3 | -3.68 | 39.86 | -19.6 | 8.9 | -66.3 |
| 54593 | MFN | 1899 | TAN | Tanga. Usambara | 1209 | -4.88 | 38.52 | -20.6 | 6.5 | -63.6 |
| 54595 | MFN | 1899 | TAN | Tanga. Usambara | 1209 | -4.88 | 38.52 | -20.4 | 6.2 | -64.8 |
| 54662 | MFN | 1899 | TAN | Tanga. Usambara | 1209 | -4.88 | 38.52 | -20.2 | 8.6 | -65.7 |
| 67053 | MFN | 1910 | TAN | Mikindani. Mtwara | 8 | -10.28 | 40.11 | -21.5 | 8.7 | -88.9 |
| 67064 | MFN | 1899 | TAN | Tanga. Usambara | 1209 | -4.88 | 38.52 | -21.1 | 6.3 | -65.8 |
| 67071 | MFN | 1907 | TAN | Mkokotoni | 14 | -5.88 | 39.25 | -20.9 | 6.1 | -75.6 |
| 67072 | MFN | 1907 | TAN | Mkokotoni | 14 | -5.88 | 39.25 | -21.2 | 6.5 | -74.4 |
| 101424 | MFN | na | TAN | Dar es Salaam | 21 | -6.82 | 39.27 | -20.0 | 8.0 | -73.0 |
| 101425 | MFN | na | TAN | Dar es Salaam | 21 | -6.82 | 39.27 | -19.2 | 8.9 | -66.6 |
| 1897-1510 | MNHN | na | TAN | Tanga. Usambara | 1209 | -4.88 | 38.52 | -19.9 | 7.7 | -69.5 |
| 1911- 1565A | MNHN | 1910 | RC | Kinshasa | 350 | -4.44 | 16.17 | -21.1 | 8.7 | -75.3 |
| 1911- 1565B | MNHN | 1910 | RC | Kinshasa | 350 | -4.44 | 16.17 | -21.8 | 6.8 | -81.3 |
| 1911- 1565C | MNHN | 1910 | RC | Kinshasa | 350 | -4.44 | 16.17 | -21.3 | 9.3 | -76.0 |
| **HM** |  |  |  |  |  |  |  |  |  |  |
| 4955 | MFN | na | Gabon | Donguila | 58 | 0.21 | 9.74 | -21.5 | 5.7 | -85.5 |
| 7043 | MFN | na | CAM | Bonge | 575 | 2.87 | 11.17 | -21.5 | 8.9 | -72.8 |
| 9008 | MFN | 1895 | Togo | Misahöhe | 295 | 6.93 | 0.60 | -21.6 | 5.0 | -80.6 |
| 9009 | MFN | 1895 | Togo | Misahöhe | 295 | 6.93 | 0.60 | -21.5 | 5.7 | -75.0 |
| 9012 | MFN | na | Gabon | Gabon | 214 | -0.38 | 11.80 | -21.8 | 4.9 | -88.0 |
| 54205 | MFN | na | CAM | Limbe | 36 | 4.02 | 9.20 | -22.1 | 5.6 | -89.7 |
| 54206 | MFN | na | CAM | Limbe | 36 | 4.02 | 9.20 | -22.0 | 6.2 | -89.0 |
| 54207 | MFN | na | CAM | Limbe | 36 | 4.02 | 9.20 | -22.2 | 5.3 | -93.9 |
| 54600 | MFN | 1899 | CAM | Limbe | 36 | 4.02 | 9.20 | -21.7 | 7.1 | -80.9 |
| 54601 | MFN | 1902 | CAM | Limbe | 36 | 4.02 | 9.20 | -21.1 | 10.4 | -67.3 |
| 54604 | MFN | 1898 | CAM | Limbe | 36 | 4.02 | 9.20 | -22.2 | 6.9 | -86.3 |
| 54696 | MFN | 1899 | CAM | Limbe | 36 | 4.02 | 9.20 | -21.9 | 6.5 | -84.9 |
| 54697 | MFN | na | CAM | Yaounde | 726 | 3.87 | 11.52 | -20.9 | 9.1 | -65.1 |
| 54698 | MFN | 1898 | CAM | Limbe | 36 | 4.02 | 9.20 | -22.4 | 7.4 | -85.8 |
| 67120 | MFN | 1902 | CAM | Limbe | 36 | 4.02 | 9.20 | -22.1 | 7.2 | -82.7 |
| 67121 | MFN | 1902 | CAM | Limbe | 36 | 4.02 | 9.20 | -22.5 | 6.0 | -101.4 |
| 67122 | MFN | 1907 | CAM | Bipindi | 78 | 3.08 | 10.41 | -22.5 | 7.6 | -83.9 |
| 67123 | MFN | 1902 | CAM | Limbe | 36 | 4.02 | 9.20 | -22.3 | 6.2 | -82.0 |
| 67125 | MFN | 1902 | CAM | Limbe | 36 | 4.02 | 9.20 | -22.2 | 6.5 | -89.8 |
| 67126 | MFN | 1898 | CAM | Limbe | 36 | 4.02 | 9.20 | -22.1 | 5.6 | -91.9 |
| 67127 | MFN | 1897 | CAM | Cameroon | 710 | 5.58 | 12.71 | -21.7 | 6.5 | -85.5 |
| 67128 | MFN | 1907 | CAM | Bipindi | 78 | 3.08 | 10.41 | -22.1 | 7.7 | -87.3 |
| 67129 | MFN | 1910 | CAM | Bipindi | 78 | 3.08 | 10.41 | -21.9 | 9.0 | -74.1 |
| 67130 | MFN | 1897 | CAM | Cameroon | 710 | 5.58 | 12.71 | -21.9 | 6.4 | -82.6 |
| 67131 | MFN | 1907 | CAM | Bipindi | 78 | 3.08 | 10.41 | -22.0 | 9.1 | -66.2 |
| 67132 | MFN | 1902 | CAM | Limbe | 36 | 4.02 | 9.20 | -22.6 | 7.1 | -82.0 |
| 67133 | MFN | 1908 | CAM | Cameroon | 710 | 5.58 | 12.71 | -21.6 | 7.2 | -82.0 |
| 67134 | MFN | 1906 | CAM | Bipindi | 78 | 3.08 | 10.41 | -21.6 | 8.7 | -81.8 |
| 67135 | MFN | 1899 | CAM | Limbe | 36 | 4.02 | 9.20 | -21.8 | 6.3 | -101.2 |
| 67157 | MFN | 1901 | CAM | Bipindi | 78 | 3.08 | 10.41 | -22.2 | 10.1 | -79.5 |
| **LA** |  |  |  |  |  |  |  |  |  |  |
| 6758 | MFN | na | Togo | Bismarckburg | 621 | 8.04 | 1.12 | -21.6 | 4.7 | -82.2 |
| 6759 | MFN | na | Togo | Bismarckburg | 621 | 8.04 | 1.12 | -22.9 | 6.9 | -82.8 |
| 6760 | MFN | na | Togo | Bismarckburg | 621 | 8.04 | 1.12 | -22.0 | 6.3 | -75.1 |
| 54982 | MFN | 1891 | CAM | Buea | 963 | 4.16 | 9.23 | -22.2 | 7.7 | -87.6 |
| 67158 | MFN | 1908 | TAN | Tanga. Amani | 905 | -5.10 | 38.63 | -20.3 | 6.4 | -90.8 |
| 67159 | MFN | 1938 | CAM | Victoria | 24 | 4.01 | 9.22 | -23.7 | 6.4 | -97.6 |
| 67160 | MFN | 1938 | CAM | Victoria | 24 | 4.01 | 9.22 | -23.2 | 6.4 | -90.3 |
| 67161 | MFN | 1938 | CAM | Victoria | 24 | 4.01 | 9.22 | -23.5 | 6.8 | -93.9 |
| 67162 | MFN | 1938 | CAM | Victoria | 24 | 4.01 | 9.22 | -23.1 | 7.8 | -90.1 |
| 67163 | MFN | 1938 | CAM | Victoria | 24 | 4.01 | 9.22 | -23.1 | 6.6 | -84.9 |
| 67164 | MFN | 1938 | CAM | Victoria | 24 | 4.01 | 9.22 | -23.0 | 7.4 | -88.1 |
| 67165 | MFN | 1938 | CAM | Victoria | 24 | 4.01 | 9.22 | -25.6 | 6.4 | -100.8 |
| 67166 | MFN | 1938 | CAM | Victoria | 24 | 4.01 | 9.22 | -23.5 | 6.1 | -90.9 |
| 67167 | MFN | 1938 | CAM | Victoria | 24 | 4.01 | 9.22 | -22.6 | 6.8 | -91.3 |
| 67168 | MFN | 1938 | CAM | Victoria | 24 | 4.01 | 9.22 | -23.2 | 6.6 | -87.9 |
| 2011- 804 | MNHN | na | DRC | Eidolon land | 1712 | -9.34 | 25.72 | -23.2 | 5.1 | -87.5 |
| 2011- 805 | MNHN | na | DRC | Eidolon land | 1712 | -9.34 | 25.72 | -22.3 | 5.5 | -83.0 |
| **RA** |  |  |  |  |  |  |  |  |  |  |
| 2553 | MFN | na | SAF | Cap of Hope | 0 | -34.36 | 18.47 | -19.1 | 6.6 | -71.7 |
| 5603 | MFN | na | TAN | Tanga | 14 | -5.07 | 39.10 | -20.7 | 13.0 | -84.8 |
| 10128 | MFN | na | TAN | Tanga | 24 | -5.07 | 39.10 | -20.7 | 8.0 | -83.8 |
| 10129 | MFN | na | TAN | Tanga | 24 | -5.07 | 39.10 | -21.0 | 9.3 | -83.3 |
| 10130 | MFN | na | TAN | Tanga | 24 | -5.07 | 39.10 | -20.7 | 9.7 | -69.3 |
| 10231 | MFN | 1893 | TAN | Tanga | 14 | -5.07 | 39.10 | -19.1 | 8.8 | -72.6 |
| 10239 | MFN | na | CAM | Yaounde | 726 | 3.87 | 11.52 | -22.4 | 6.5 | -96.6 |
| 54199 | MFN | na | TAN | Bukoba. Kagera | 1148 | -1.32 | 31.81 | -20.3 | 9.4 | -83.0 |
| 54200 | MFN | na | TAN | Bukoba. Kagera | 1148 | -1.32 | 31.81 | -19.9 | 9.9 | -75.5 |
| 54369 | MFN | 1905 | TAN | Tanga | 24 | -5.07 | 39.10 | -20.6 | 7.7 | -74.3 |
| 54522 | MFN | na | TAN | Bukoba. Kagera | 1148 | -1.32 | 31.81 | -20.7 | 7.2 | -85.2 |
| 54691 | MFN | na | CAM | Bipindi | 78 | 3.08 | 10.41 | -21.8 | 11.5 | -83.8 |
| 54699 | MFN | 1906 | CAM | Bipindi | 78 | 3.08 | 10.41 | -22.4 | 11.0 | -83.9 |
| 54700 | MFN | 1905 | CAM | Limbe | 36 | 4.02 | 9.20 | -22.1 | 8.3 | -89.0 |
| 67226 | MFN | 1911 | ANG | Cabinda | 95 | -5.02 | 12.35 | -22.2 | 8.4 | -83.1 |
| 67227 | MFN | na | Kenya | Kibwezi | 899 | -2.41 | 37.97 | -20.2 | 8.3 | -73.9 |
| 67230 | MFN | na | TAN | Bukoba. Kagera | 1148 | -1.32 | 31.81 | -20.8 | 9.1 | -73.9 |
| 67231 | MFN | na | TAN | Bukoba. Kagera | 1148 | -1.32 | 31.81 | -20.5 | 8.5 | -81.2 |
| 67232 | MFN | 1898 | TAN | Zanzibar | 33 | -6.17 | 39.20 | -20.8 | 7.3 | -75.1 |
| 67239 | MFN | na | TAN | Bukoba. Kagera | 1148 | -1.32 | 31.81 | -20.8 | 7.1 | -80.5 |
| 67240 | MFN | na | TAN | Bukoba. Kagera | 1148 | -1.32 | 31.81 | -20.8 | 6.8 | -74.5 |
| 1895- 390 | MNHN | na | TAN | Tanga. Usambara | 1209 | -4.88 | 38.52 | -20.4 | 6.8 | -74.9 |
| 1895- 391 | MNHN | na | TAN | Tanga. Usambara | 1209 | -4.88 | 38.52 | -20.7 | 6.9 | -75.2 |
| 1895- 392 | MNHN | na | TAN | Tanga. Usambara | 1209 | -4.88 | 38.52 | -20.1 | 7.5 | -82.2 |
| 1895- 393 | MNHN | na | TAN | Tanga. Usambara | 1209 | -4.88 | 38.52 | -20.1 | 6.8 | -75.4 |
| 1897-1511A | MNHN | na | TAN | Tanga. Usambara | 1209 | -4.88 | 38.52 | -20.5 | 6.9 | -85.6 |
| 1897- 1511B | MNHN | na | TAN | Tanga. Usambara | 1209 | -4.88 | 38.52 | -20.6 | 9.5 | -81.0 |
| 1911- 725A | MNHN | 1909 | TAN | Tanga | 14 | -5.07 | 39.10 | -20.5 | 6.5 | -74.7 |
| 1911- 725B | MNHN | 1909 | TAN | Tanga | 14 | -5.07 | 39.10 | -20.8 | 7.0 | -74.1 |
| 1911- 725C | MNHN | 1909 | TAN | Tanga | 14 | -5.07 | 39.10 | -20.1 | 7.6 | -70.4 |
| 1911- 725D | MNHN | 1909 | TAN | Tanga | 14 | -5.07 | 39.10 | -20.7 | 7.2 | -64.8 |
| 1911- 726A | MNHN | 1909 | TAN | Tanga | 14 | -5.07 | 39.10 | -20.5 | 8.7 | -63.7 |
| 1911- 726B | MNHN | 1909 | TAN | Tanga | 14 | -5.07 | 39.10 | -20.7 | 8.1 | -67.0 |
| 1913- 22 | MNHN | 1905 | TAN | Tanga | 14 | -5.07 | 39.10 | -21.7 | 6.9 | -84.4 |
